# Multicohort external validation of *Anaeroglobus* as a candidate salivary biomarker for childhood caries

**DOI:** 10.3389/fcimb.2026.1850479

**Published:** 2026-08-14

**Authors:** Jing Wang, Ye Tao, Hua-don Meng, Xiong-fen Zhu, Xiu-min Xu, Da-wei Mi

**Affiliations:** 1 Department of Pediatric Dentistry, Hefei Stomatological Hospital, Hefei, Anhui, China; 2 Anhui Institute of Medicine Affiliated Stomatological Hospital, Hefei, Anhui, China; 3 Anhui Institute of Medicine, Hefei, Anhui, China; 4 Department of Stomatology, Suzhou Hospital of Anhui Medical University (Suzhou Municipal Hospital of Anhui Province), Suzhou, Anhui, China

**Keywords:** *Anaeroglobus*, childhood caries, dental plaque, external validation, salivary microbiome, single-cell, transcriptome

## Abstract

**Objective:**

To evaluate the cross-cohort reproducibility of *Anaeroglobus* as a candidate salivary microbiome signal for childhood caries and to place this signal within plaque community, functional, and host-response contexts.

**Methods:**

Public pediatric salivary 16S rRNA cohorts were analyzed using a discovery–primary external validation–second validation framework. Genus-level features were assessed after CLR transformation and covariate adjustment, with plaque microbiome, plaque transcriptome, and deep-caries dental pulp single-cell datasets used as supporting evidence layers.

**Results:**

In the discovery cohort, *Lactobacillus* was associated with caries status and *Anaeroglobus* with total caries burden. In the primary external cohort, *Anaeroglobus* and *Lactobacillus* remained positively associated with caries [OR = 3.452 (95% CI: 1.851–6.449) and OR = 2.118 (95% CI: 1.049–4.282), respectively]. In the second validation cohort, Anaeroglobus remained directionally consistent [OR = 3.009 (95% CI: 1.241–7.312)]. The primary external microbiome model showed moderate discrimination (AUC = 0.782; approximate 95% CI: 0.703–0.861). Exploratory pooled-effect synthesis supported positive directional consistency, while the supporting plaque and multi-omics layers indicated broader community restructuring, carbohydrate/oxidative-stress adaptation, and host immune–stromal remodeling.

**Conclusions:**

*Anaeroglobus* represents a directionally reproducible candidate salivary risk-related signal rather than a direct mechanistic or immediately diagnostic biomarker. The 36-sample second validation cohort should be interpreted as supportive age-extrapolation evidence, and the pooled synthesis should be interpreted as exploratory; further prospective, paired saliva–plaque, and multimodal validation is required.

## Introduction

Childhood caries, particularly early childhood caries (ECC), remains one of the most prevalent oral diseases worldwide, and the burden of untreated caries remains substantial (Kassebaum et al., 2015; Peres et al., 2019; World Health Organization, 2022). The World Health Organization (WHO) implementation guidance also emphasizes prevention-oriented and early interventions for childhood caries control (World Health Organization, 2020). At the etiologic level, explanations for caries have shifted from traditional single-pathogen views toward frameworks centered on dental plaque biofilms, community imbalance, and ecological pressure (Marsh, 1994; Marsh, 2006; Rosier et al., 2014). This ecological perspective has been further refined by later conceptual work (Takahashi and Nyvad, 2011; Simón-Soro and Mira, 2015; Nyvad and Takahashi, 2020), and maintenance of oral microbial resilience is now considered important for disease prevention (Rosier et al., 2018). With the rapid development of high-throughput sequencing and oral multi-omics, increasing effort has been directed toward identifying candidate microbial genera, building risk-prediction models, and evaluating whether microbiome-associated signals can be replicated across cohorts (Zarco et al., 2012; Hemadi et al., 2017; Xiao et al., 2020). However, the pediatric oral microbiome itself shows clear age-dependent developmental features and substantial between-cohort heterogeneity (Gomez and Nelson, 2017; D'Agostino et al., 2022), with ecological succession continually shaped by postnatal factors and developmental stage (Dzidic et al., 2018; Kahharova et al., 2020). Earlier studies have also provided important background on the normal oral microbiota of children and on host genetic influences on oral microbial composition (Aas et al., 2005; Gomez et al., 2017).

Accordingly, current ECC research increasingly emphasizes multispecies biofilms and niche-based interpretation rather than single-genus determinism (Hajishengallis et al., 2017). On this basis, the present study follows a three-layer saliva-based path of discovery, primary external validation, and second independent external validation to evaluate the directional consistency and external reproducibility of Anaeroglobus and related candidate genera. Same-ecological plaque evidence, plaque transcriptomics, and deep-caries dental pulp single-cell findings are introduced only as supporting layers to contextualize community background, functional background, and host-response background, rather than to replace the main saliva-based analysis.

## Materials and methods

### Study design and data sources

**Figure 1:**
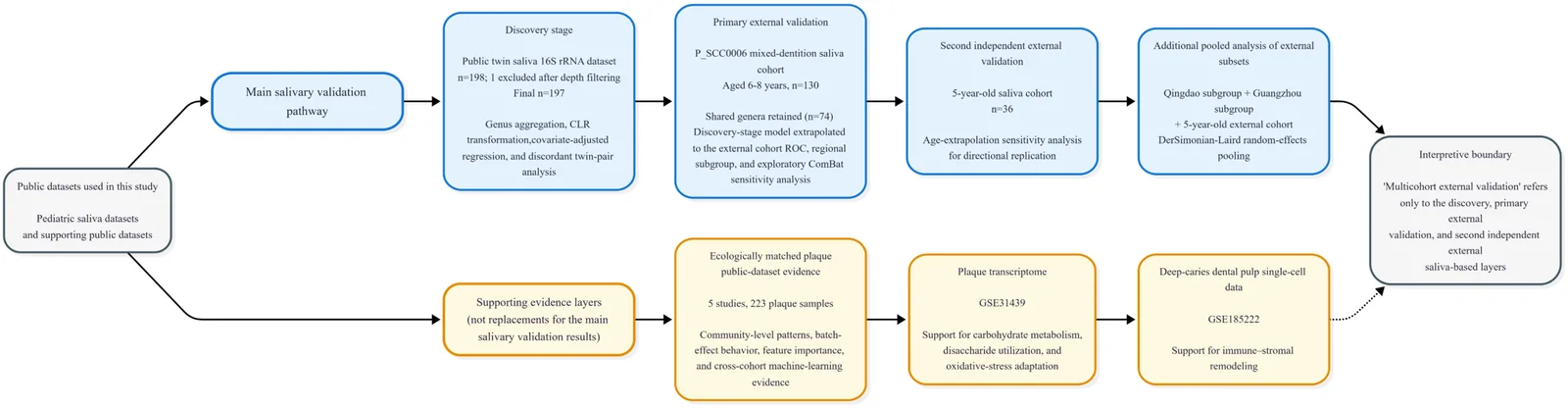
Study design and cohort flowchart.

This study comprised three components. First, the completed saliva-based multicohort analysis included a public twin saliva discovery cohort (198 samples, 197 retained after depth filtering) (Kasimoglu et al., 2020), a public mixed-dentition saliva cohort for primary external validation (P_SCC0006, n=130) (Li et al., 2021), and a public saliva cohort of 5-year-old children for second independent external validation (n=36) (Yang et al., 2023). The overall study design and cohort flowchart are shown in **Figure 1**. Because the second independent validation cohort included only 36 children, it was prespecified as a supportive age-extrapolation validation layer and was not assigned the same evidentiary weight as the 130-sample primary external validation cohort.

Second, same-ecological dental plaque public-database evidence was incorporated from a published cross-cohort plaque integration study including five ECC plaque studies and 223 plaque samples (Khan et al., 2024). These data were used to supplement beta-diversity patterns, batch-effect behavior, feature-importance patterns, and cross-cohort machine-learning performance. It should be emphasized that four of the five plaque cohorts integrated by Khan et al. can be downloaded from their respective Sequence Read Archive (SRA) repositories, whereas one cohort requires author contact; therefore, the present manuscript does not claim to have completed a unified raw-sequence reprocessing workflow or new individual-level PCoA/PERMANOVA, LEfSe, or PICRUSt2 analyses for all plaque cohorts.

Third, functional and host-response support was added from the public plaque transcriptome dataset GSE31439 (Peterson et al., 2014) and the deep-caries dental pulp single-cell dataset GSE185222 (Opasawatchai et al., 2022). Both layers were explicitly treated as supporting evidence and were not used to replace the completed main external validation results.

### Statistical analysis and methodological boundary of the supporting evidence layer

In the discovery cohort, total caries burden was defined as the sum of dmfs-index and DMFS-index, and samples with a total caries burden >0 were classified as caries-positive. ASVs were aggregated to the genus level, filtered by detection frequency, transformed using centered log-ratio methods (Aitchison, 1982; Gloor et al., 2017), and analyzed in regression models adjusted for age, sex, zygosity, and dentition stage. Discordant twin pairs were further analyzed within families, and TwinID was used as the clustering unit to account for family relatedness. Only genera shared across cohorts were retained for the primary and second external validation stages, and exploratory regional subgroup analyses together with ComBat-based sensitivity analysis were performed (Johnson et al., 2007). For the exploratory pooled-effect analysis across completed validation subsets, odds ratios were log-transformed, standard errors were back-calculated from the reported 95% confidence intervals, and pooled effects were estimated using a DerSimonian–Laird random-effects model (DerSimonian and Laird, 1986). Multiple-testing control was based on the Benjamini–Hochberg procedure (Benjamini and Hochberg, 1995). The supporting evidence layer relied on already published and reproducible quantitative results from public databases and was used only to contextualize the same-ecological community background, the functional background, and the host-response background of the main saliva findings. Throughout this manuscript, the term “multicohort external validation” refers only to the discovery, primary external, and second independent external saliva validation layers.

To improve reproducibility and clarify model assumptions, genus-level features were retained only when they were detectable after prevalence filtering and could be aligned across the discovery and validation feature spaces; zero handling was performed before CLR transformation using a small pseudocount consistently within each cohort. The primary analyses were interpreted as association and validation analyses rather than causal inference. Because microbiome data are sparse, compositional, and potentially zero-inflated, the regional subgroup analysis, ComBat-based sensitivity analysis, and leave-one-out pooled-effect analysis were used as robustness checks. The pooled-effect analysis was considered exploratory because the Qingdao and Guangzhou subsets originated from the same parent validation cohort and therefore could not be treated as fully independent studies.

For the 36-sample second validation cohort, a *post-hoc* precision check was additionally performed by back-calculating log(OR) standard errors from the reported 95% CIs. This analysis was used only to quantify estimate imprecision and did not upgrade the evidentiary level of the small cohort.

Model discrimination in the primary external-validation cohort was summarized using the AUC and its approximate 95% CI based on the 65 caries and 65 caries-free samples. Because no clinically prespecified probability threshold was available for the public validation reanalysis, threshold-dependent indices such as sensitivity, specificity, positive predictive value, negative predictive value, calibration slope, and decision-curve analysis were not treated as definitive primary results and are instead listed as requirements for future individual-level validation.

## Results

### Cohort composition and baseline characteristics

A total of 327 pediatric saliva samples were included in the main analysis, comprising 197 samples in the discovery cohort and 130 samples in the primary external validation cohort; an additional 36 samples from the 5-year-old cohort were included for second independent external validation. In the discovery cohort, 151 children had caries and 46 were caries-free, involving 99 twin pairs, of which 18 pairs were discordant for caries status and therefore informative for within-family analysis. The primary external validation cohort consisted entirely of mixed-dentition saliva samples from children aged 6–8 years, including 65 caries cases and 65 controls. Baseline characteristics and alpha-diversity comparisons in the discovery cohort are presented in **Table 1**.

**Table 1:** Baseline characteristics and alpha diversity in the discovery cohort by caries status.

Variable	Caries-free (n=46)	Caries (n=151)	*P*
Age (years)	10.50 (9.00, 12.75)	9.00 (7.00, 12.00)	0.016
Plaque index	0.00 (0.00, 0.33)	0.16 (0.00, 0.38)	0.017
Gingival index	0.00 (0.00, 0.08)	0.00 (0.00, 0.16)	0.032
Shannon index	2.88 (2.57, 3.13)	2.84 (2.56, 3.11)	0.547
Observed genera	42.00 (36.00, 48.00)	41.00 (35.00, 47.00)	0.623
Female, n (%)	23 (50.0)	73 (48.3)	0.842

### Key caries-associated genera in the discovery cohort

**Figure 2:**
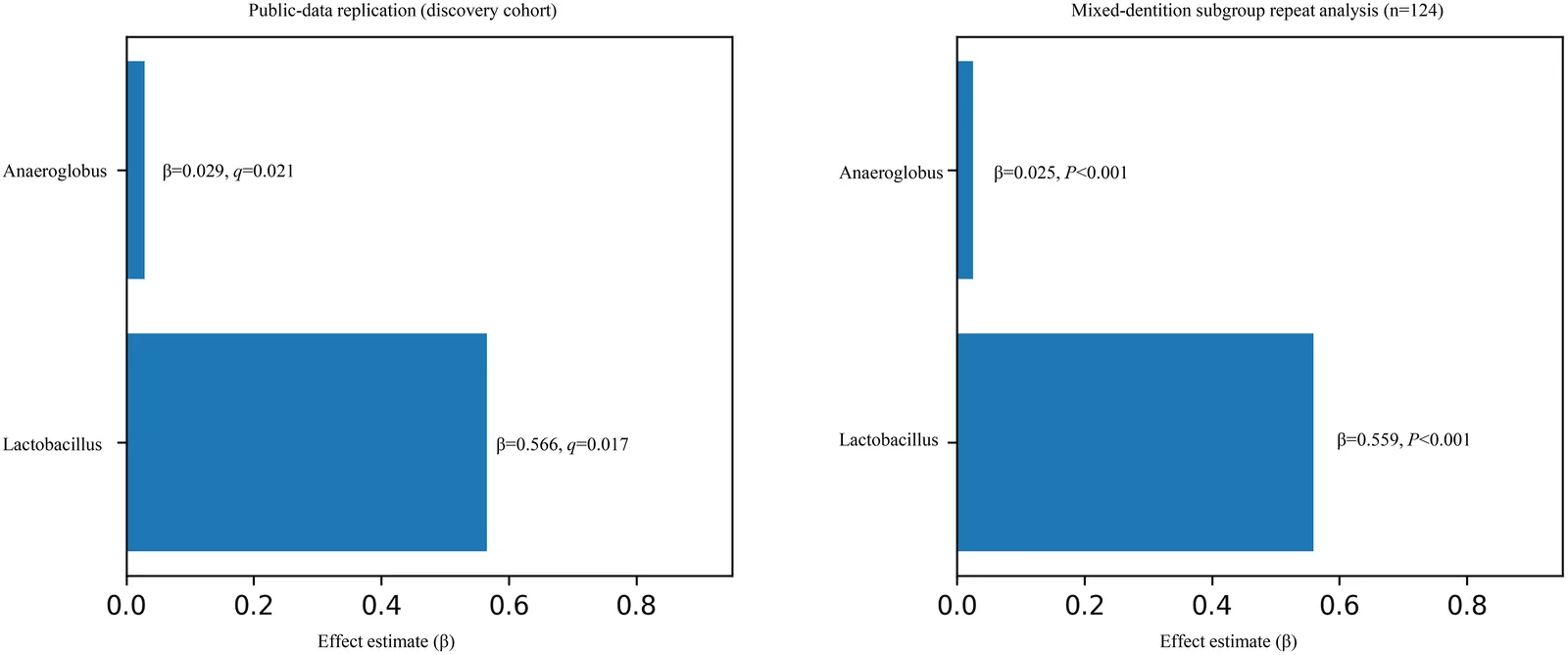
Discovery-stage effect estimates of the key genera.

After adjustment for age, sex, zygosity, and dentition stage with robust standard errors clustered by Twin ID, Lactobacillus remained independently associated with caries status (β=0.566, *q* = 0.017), Candidatus_Saccharibacteria_bacterium_UB2523 also showed a positive association (β=0.398, *q* = 0.013), and Anaeroglobus was positively associated with total caries burden (β=0.029, *q* = 0.021). In the 18 discordant twin pairs, no genus remained significant after FDR correction, indicating that individual genus effects were limited under strict within-family constraints. These discovery-stage results are summarized in **Table 2** and visualized in **Figure 2**.

**Table 2:** Key genera in the discovery stage and summary of within-family paired analysis.

Genus	Discovery-stage endpoint/effect	Statistical result	Within-family paired result
Lactobacillus	Caries status	β=0.566, *q* = 0.017	Not significant after FDR
Candidatus_Saccharibacteriabacterium_UB2523	Caries status	β=0.398, *q* = 0.013	Not significant after FDR
Anaeroglobus	Total caries burden	β=0.029, *q* = 0.021	Not significant after FDR

### Primary and second independent external validation results

**Figure 3:**
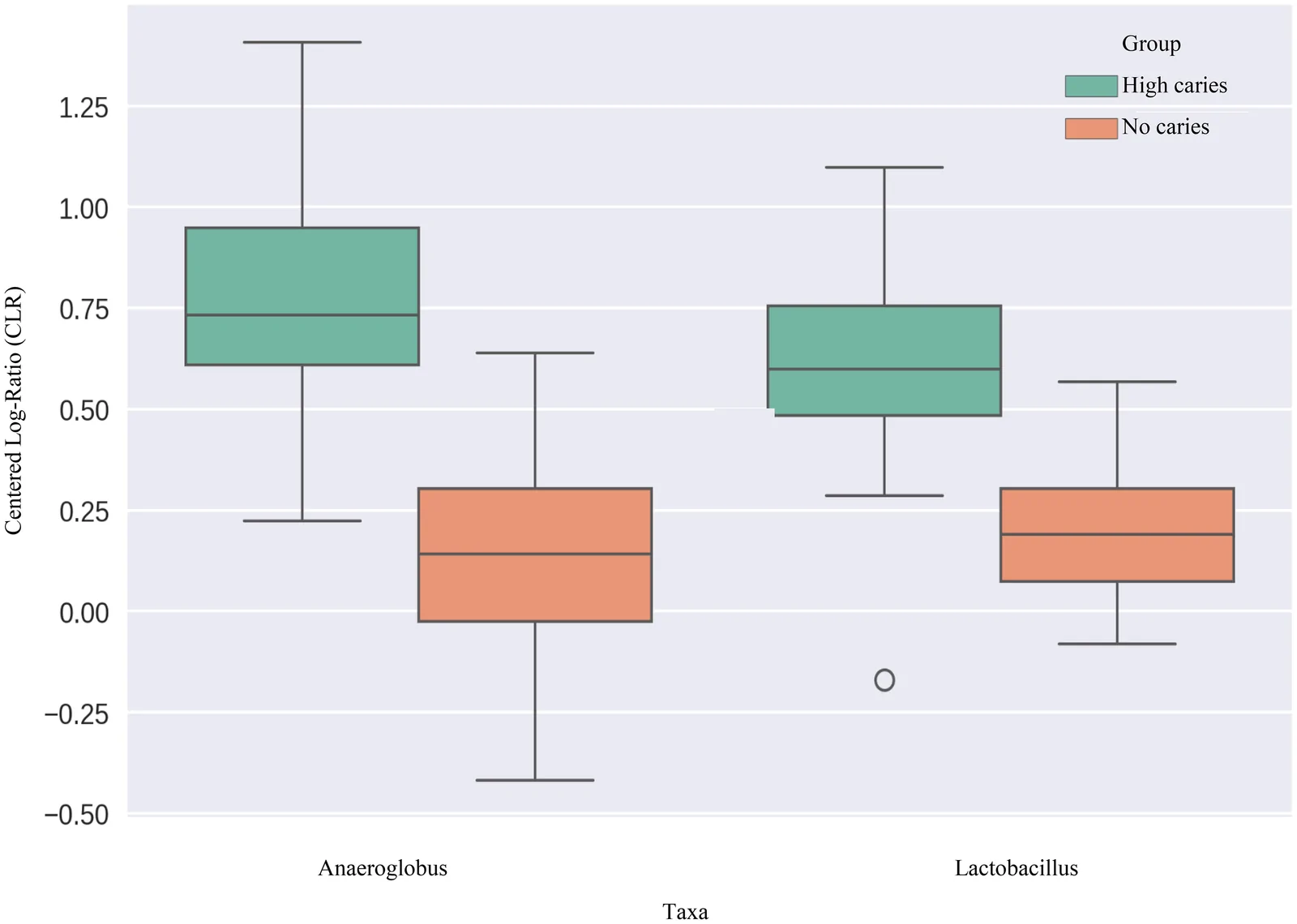
CLR abundance distributions of *Anaeroglobus* and Lactobacillus in the primary external validation cohort. The primary external validation cohort included 65 caries cases and 65 controls (n=130).

**Figure 4:**
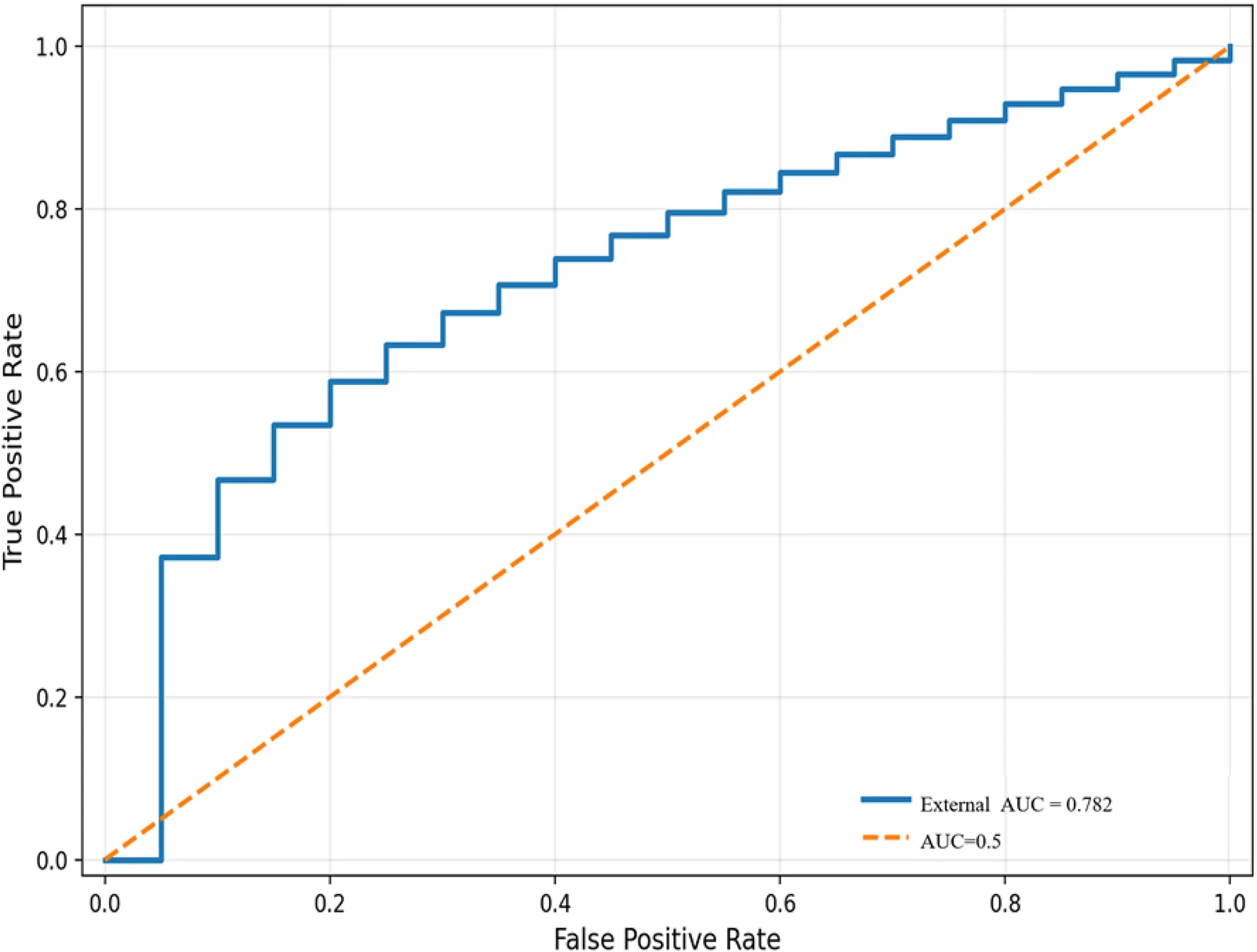
ROC curve of the microbiome model in the primary external validation cohort. AUC = 0.782; approximate 95% CI: 0.703-0.861; n=130.

**Figure 5:**
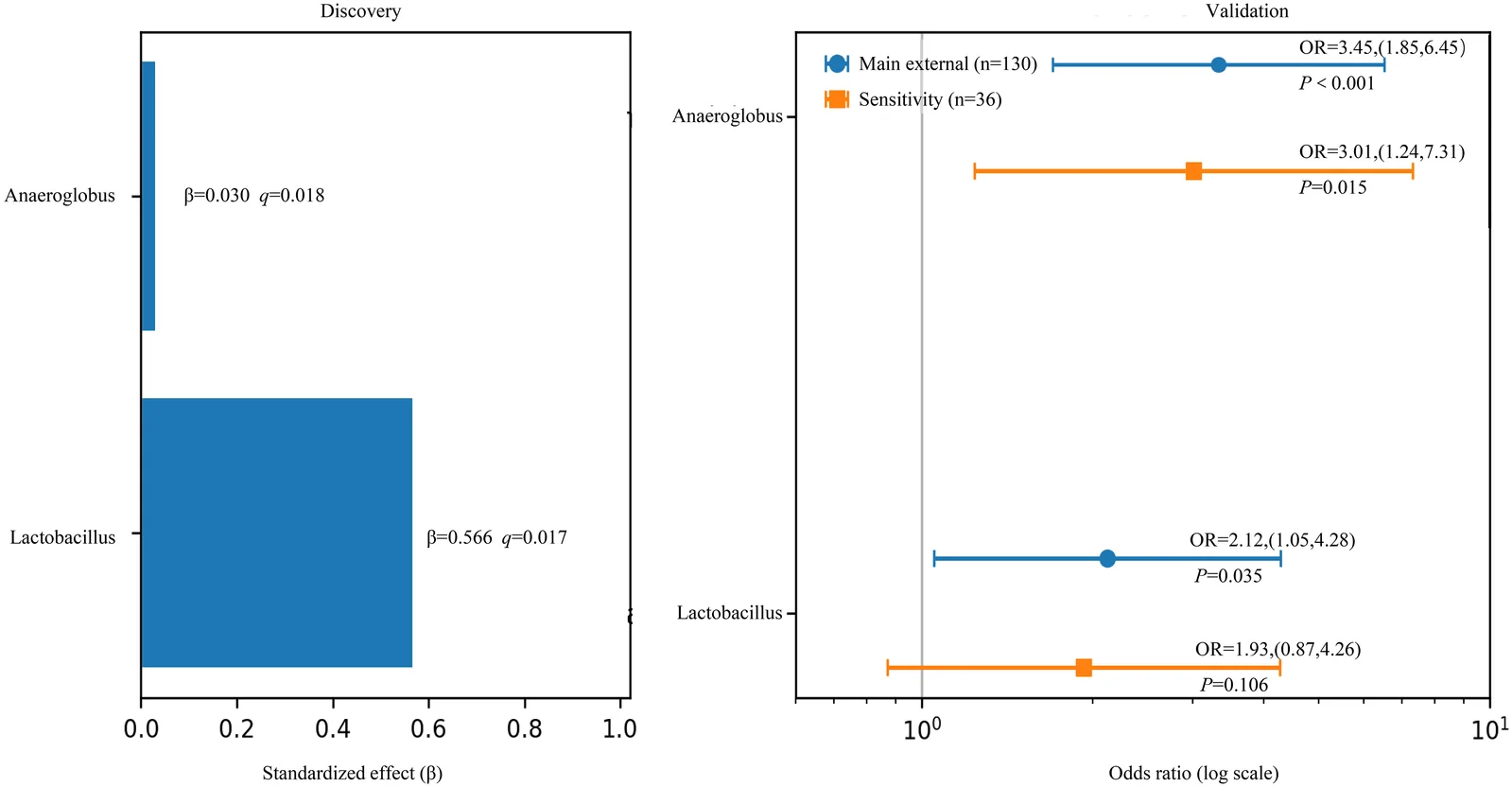
Effect estimates of the key genera across the discovery, primary external, and second independent external validation stages. External validation estimates are shown with 95% CIs; the 36-sample cohort is interpreted as supportive age-extrapolation evidence.

In the primary external validation cohort, Anaeroglobus showed higher CLR abundance in caries cases than in controls, with an adjusted OR of 3.452 (95% CI: 1.851–6.449) per one-standard-deviation increase. Lactobacillus remained directionally consistent and statistically significant, with an OR of 2.118 (95% CI: 1.049–4.282). In the second independent external validation cohort, Anaeroglobus remained directionally consistent and significant [OR = 3.009, 95% CI: 1.241–7.312; *P* = 0.015], whereas Lactobacillus remained directionally consistent but did not reach statistical significance [OR = 1.929 (95% CI: 0.866–4.262); *P* = 0.106]. These external validation results are presented in **Table 3**, with the corresponding CLR distributions, ROC performance, and stage-wise effect estimates shown in **Figures 3**–**5**. Given the limited sample size of the second independent cohort, this result should be interpreted mainly as supportive directional replication, and the width of the confidence interval should be considered when judging robustness. A *post-hoc* precision check confirmed this limitation: the back-calculated log(OR) SE for Anaeroglobus in the 36-sample cohort was 0.452, compared with 0.318 in the 130-sample primary cohort; for Lactobacillus, the corresponding SE was 0.407 and the confidence interval crossed unity. Thus, the second cohort mainly supports directionality rather than precise effect estimation. For the primary external microbiome model, the AUC was 0.782 with an approximate 95% CI of 0.703-0.861, providing additional uncertainty information beyond the point estimate.

**Table 3:** Results of the key genera in the primary and second independent external validation cohorts.

Genus	Discovery-stage endpoint/effect	Primary external validation OR (95% CI)	*P*	Second independent external validation OR (95% CI)	*P*
Lactobacillus	Caries status; β=0.566, q=0.017	2.118 (1.049–4.282)	0.035	1.929 (0.866–4.262)	0.106
Anaeroglobus	Caries burden; β=0.029, q=0.021	3.452 (1.851–6.449)	<0.001	3.009 (1.241–7.312)	0.015

### Regional subgroup and exploratory batch-effect sensitivity analysis

**Figure 6:**
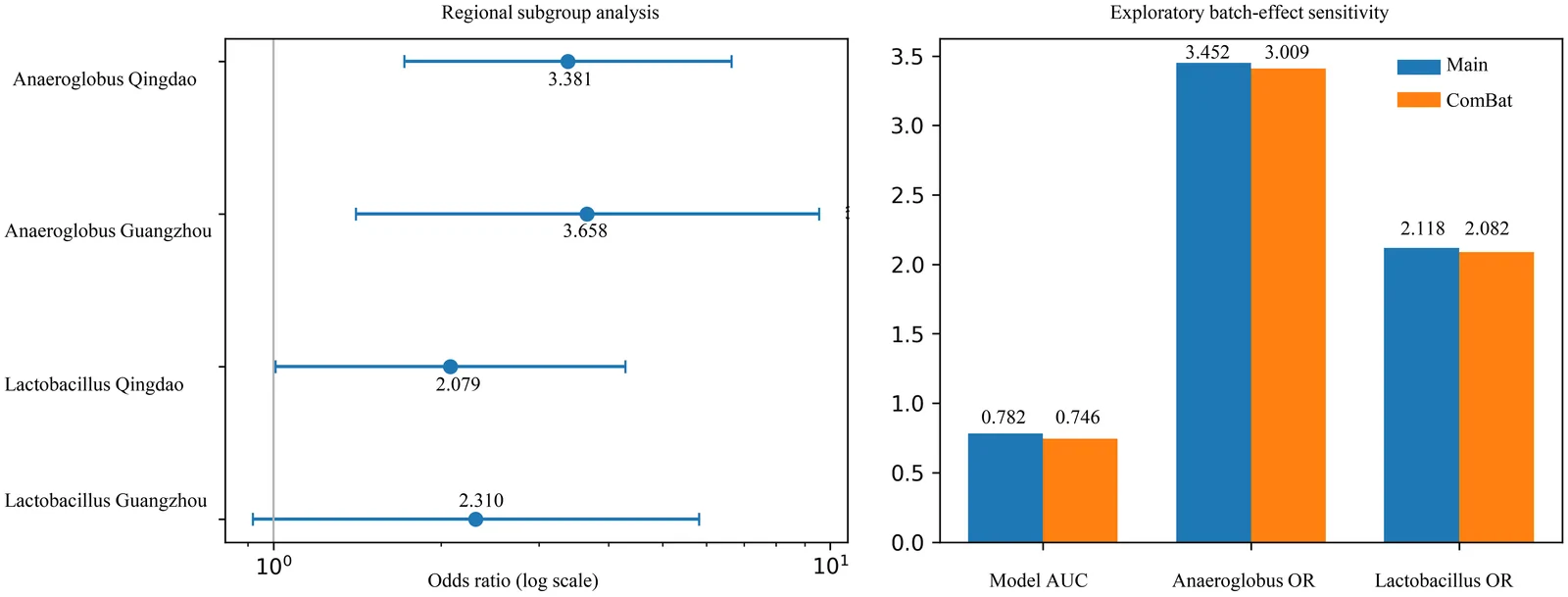
Regional subgroup and exploratory batch-effect sensitivity analysis in the primary external validation cohort. Overall n=130; ComBat-adjusted AUC = 0.746 (95% CI: 0.662-0.831).

When the primary external validation cohort was stratified by sampling region, Anaeroglobus remained directionally consistent with similar effect sizes in the Qingdao and Guangzhou subgroups; Lactobacillus also showed consistent positive associations, although the Guangzhou subgroup did not reach statistical significance. After an exploratory ComBat adjustment using region as the batch variable, the AUC changed only slightly from 0.782 to 0.746, and the direction of the Anaeroglobus and Lactobacillus effects was unchanged, suggesting that regional or batch heterogeneity did not materially alter the main interpretation. The subgroup and exploratory batch-effect sensitivity results are summarized in **Table 4** and **Figure 6**. The primary AUC uncertainty estimate (0.782, approximate 95% CI: 0.703-0.861) and the ComBat-adjusted AUC (0.746, 95% CI: 0.662-0.831) were reported to make the model-discrimination assessment more transparent.

**Table 4:** Regional subgroup and exploratory batch-effect sensitivity analysis in the primary external validation cohort.

Genus/metric	Subgroup	n	OR/AUC	95% CI	*P*
Anaeroglobus	Qingdao	96	3.381	1.721–6.649	<0.001
Anaeroglobus	Guangzhou	34	3.658	1.409–9.555	0.008
Lactobacillus	Qingdao	96	2.079	1.010–4.291	0.047
Lactobacillus	Guangzhou	34	2.310	0.920–5.820	0.075
Model AUC after ComBat	Overall cohort	130	0.746	0.661–0.831	—
Model AUC before ComBat	Overall cohort	130	0.782	0.703-0.861	—

### Same-ecological plaque public databases: community-level and feature-level support

To address the reviewer concern of “why not use dental plaque” and “how does the overall community structure differ?”, same-ecological plaque public-database evidence was incorporated. The published cross-cohort plaque study included five ECC plaque studies with 223 plaque samples (117 caries-free and 106 ECC). At the species level, the ANOSIM R values before batch correction were 0.64 in the caries-free group and 0.49 in the ECC group, indicating marked between-study community differences; after correction, these values dropped to 0.075 and 0.091, respectively, demonstrating that batch effects strongly influence overall community-level interpretation. At the feature level, the average cross-cohort random-forest AUROC was approximately 0.85 at the species level and 0.83 at the genus level, suggesting that same-ecological plaque data are more suitable for risk discrimination. However, the same study also showed that no single genus consistently ranked highest across all datasets, and the meta-analytic genus-level signal more consistently favored Alloprevotella and Megasphaera. This does not contradict our saliva-based result, but instead places Anaeroglobus within a broader plaque-level ecological background in which community restructuring is more prominent than any single-genus main effect. These community-level and feature-level findings are summarized in **Table 5**.

**Table 5:** Community-level and feature-level support from same-ecological cross-cohort plaque public databases.

Item	Result	Interpretation
Number of included studies	5	Same-ecological ECC plaque public databases
Total samples	223 (117 caries-free, 106 ECC)	Basis for cross-cohort integration
β-diversity ANOSIM R before correction	0.64 in caries-free; 0.49 in ECC	Marked between-study community differences
β-diversity ANOSIM R after correction	0.075 in caries-free; 0.091 in ECC	Batch effects markedly affect community interpretation
Average RF AUROC	0.85 at species level; 0.83 at genus level	Plaque models show relatively good discrimination
More stable genera in pooled-effect analysis	Alloprevotella, Megasphaera	Plaque evidence better captures community-level cariogenic context

### Functional support from the plaque transcriptome

To address the lack of functional evidence, we incorporated the public plaque transcriptome dataset GSE31439; this dataset included 38 plaque samples from 19 twin pairs and identified nearly 60 functionally core taxa in supragingival plaque. Carbohydrate-utilization transcripts accounted for approximately 10% of total transcripts, and monosaccharide/disaccharide metabolism-related transcripts accounted for about 15%, with disaccharide metabolism being approximately twice as abundant as monosaccharide metabolism; lactose and galactose metabolism were particularly prominent (Peterson et al., 2014; Grier et al., 2021).

**Figure 7:**
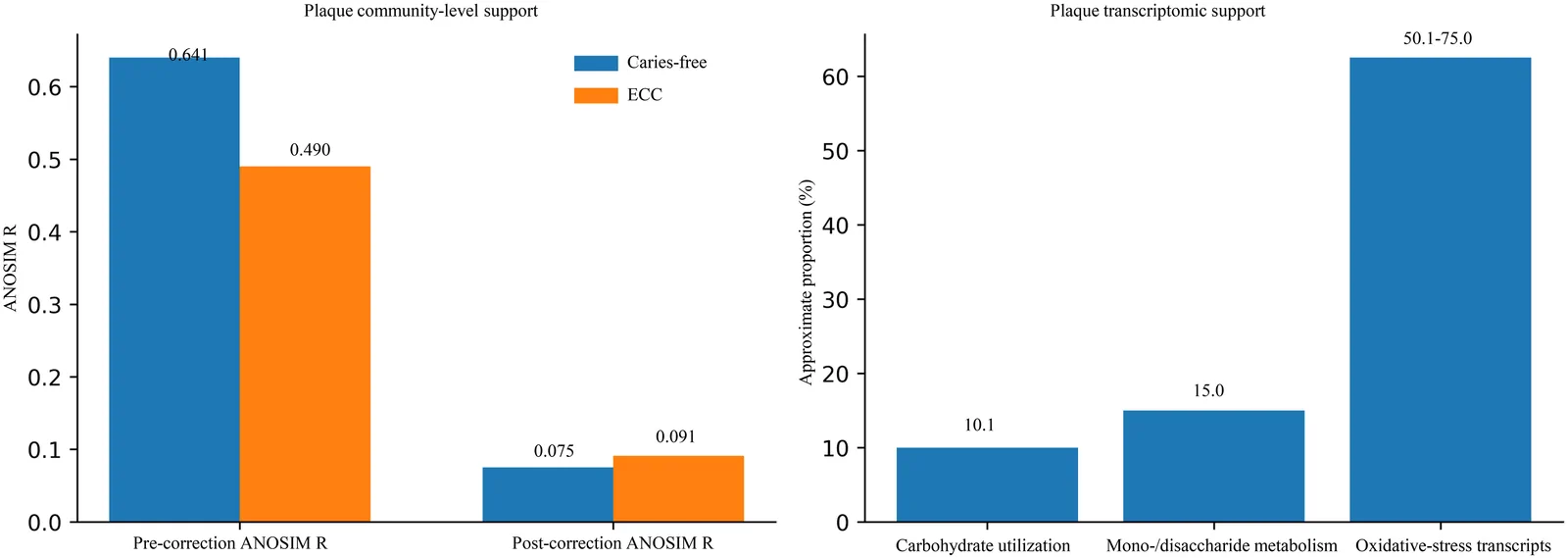
Quantitative summary of same-ecological plaque community-level and transcriptomic functional support.

In addition, oxidative-stress-related transcripts accounted for 50%-75% of stress-response transcripts, indicating that oxidative stress is a major pressure source within plaque biofilms; superoxide-dismutase-related transcripts represented about 22% of oxidative-stress transcripts and approximately 0.4% of total transcripts (Peterson et al., 2014). These results support the view that the observed microbiome signals in childhood caries arise within a broader background of carbohydrate metabolism, acid adaptation, and biofilm stabilization (Peterson et al., 2014; Wang et al., 2019; Grier et al., 2021), but they do not directly prove an Anaeroglobus-specific functional dominance. The transcriptomic functional support is summarized in **Table 6**, and the integrated quantitative summary combining plaque community-level and transcriptomic evidence is shown in **Figure 7**.

**Table 6:** Functional-layer support from the plaque transcriptome.

Functional item	Public-database result	Relevance to the present study
Dataset	GSE31439; 19 twin pairs; 38 plaque samples	Same-ecological functional support
Functionally core taxa	~60 taxa	Suggests a stable functional core in plaque
Carbohydrate utilization	~10% of all transcripts	Supports a cariogenic carbohydrate-metabolism background
Mono-/disaccharide metabolism	~15% of all transcripts; disaccharides ≈ 2× monosaccharides	Supports the importance of disaccharide use in plaque
Predominant disaccharide pathways	Lactose and galactose metabolism	Consistent with sugar exposure in childhood caries
Oxidative stress	50%–75% of stress-response transcripts	Indicates continuous oxidative-pressure adaptation in plaque
SOD-related transcripts	22% of oxidative-stress transcripts; ~0.4% of all transcripts	Supports acid/oxidative stress buffering mechanisms

### Third-layer host-response support from deep-caries dental pulp single-cell data

As a third-layer host-response support, we incorporated GSE185222, which included 6,810 dental pulp cells spanning sound, enamel-caries, and deep-caries states. Published results showed that deep caries, but not enamel-stage caries, altered pulp immune-cell composition and upregulated pro-inflammatory, anti-inflammatory, and mineralization-related genes in immune and stromal cells (Opasawatchai et al., 2022). Further cell-cell communication prediction indicated enhanced interactions with macrophages in the deep-caries state (Opasawatchai et al., 2022). These observations suggest that when a cariogenic biofilm progresses toward deep carious involvement, the host side is not static but instead shows immune-stromal remodeling and increased intercellular communication (Opasawatchai et al., 2022). Importantly, this evidence supports a generic host-response background rather than a unique Anaeroglobus-specific host mechanism (**Table 7**).

**Table 7:** Third-layer host-response support from deep-caries dental pulp single-cell data.

Item	Public-database result	Relevance to the present study
Dataset	GSE185222	Third-layer support from deep-caries pulp single-cell data
Total cells	6,810 pulp cells	Covers sound, enamel-caries, and deep-caries states
Main change	Deep caries altered the immune-cell composition of the pulp	Supports host remodeling during lesion progression
Differential-expression direction	Pro-inflammatory, anti-inflammatory, and mineralization-related genes were upregulated	Indicates simultaneous inflammatory and reparative responses
Cell–cell communication	Macrophage-related interactions increased in deep caries	Supports the biological plausibility of host–microbiome coupling

### Exploratory pooled-effect analysis across completed validation subsets

**Figure 8:**
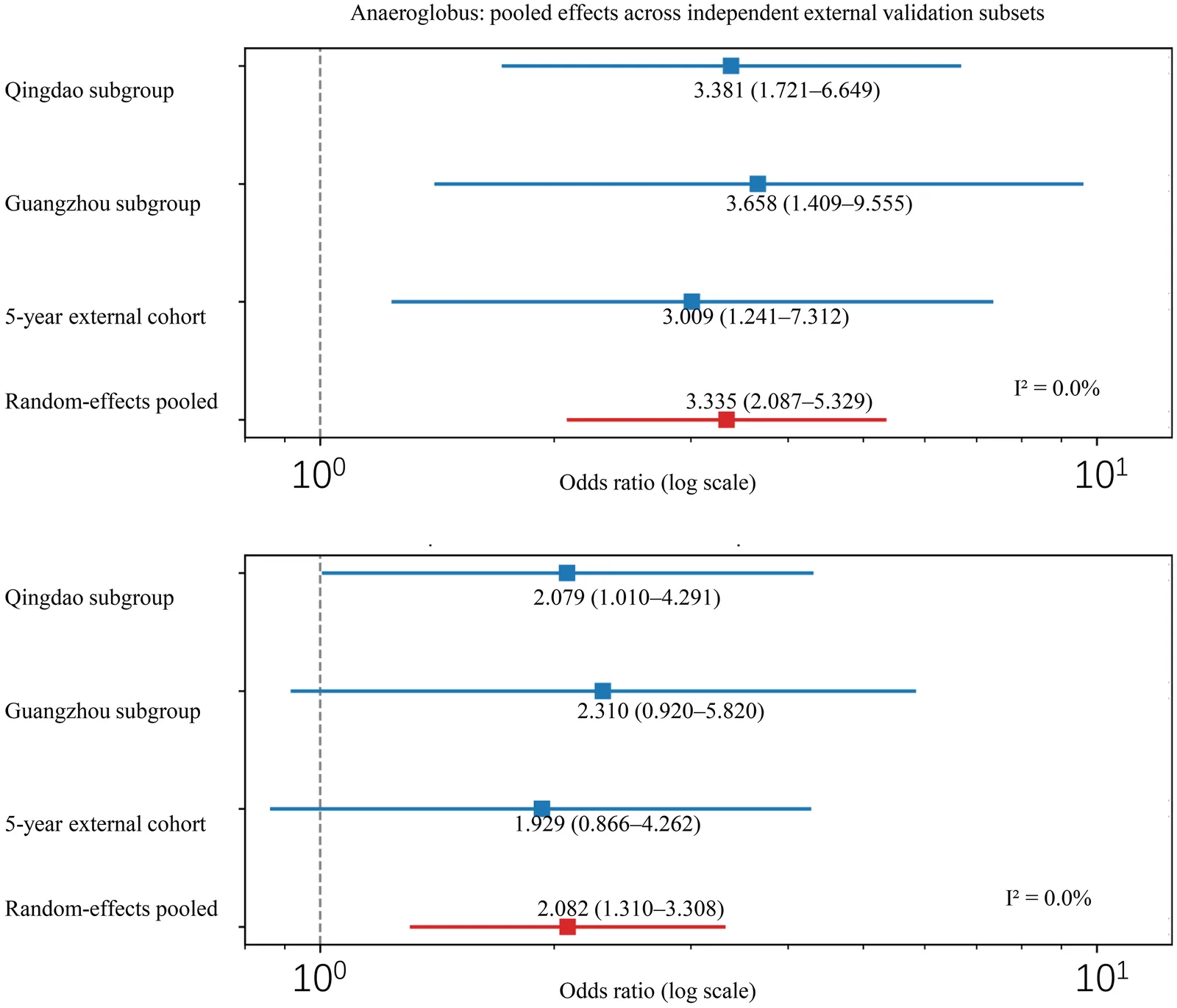
Forest plot of exploratory pooled effects across completed validation subsets. Qingdao and Guangzhou are regional subsets from the same parent cohort; therefore, the pooled estimate is descriptive and hypothesis-supporting.

Without double-counting the overall primary external validation cohort, we summarized the Qingdao subgroup, Guangzhou subgroup, and the 5-year-old external cohort as completed validation subsets and performed an exploratory random-effects pooled-effect analysis using the reported ORs and 95% CIs. Because the Qingdao and Guangzhou subsets were geographically stratified components of the same parent cohort, this synthesis was not interpreted as a fully independent multi-study pooled-effect analysis. Anaeroglobus showed a pooled OR of 3.335 (95% CI: 2.087-5.329; I²=0.0%), whereas Lactobacillus showed a pooled OR of 2.082 (95% CI: 1.310-3.308; I²=0.0%). Leave-one-out sensitivity analysis yielded a narrow pooled range for both genera, supporting the stability of the external validation signal (**Table 8**, **Figure 8**). Additional heterogeneity statistics were Q = 0.089 (P = 0.956) for Anaeroglobus and Q = 0.084 (P = 0.959) for Lactobacillus, with tau²=0 in both analyses; these values are descriptive rather than proof of full between-study independence.

**Table 8:** Exploratory pooled effects and stability analysis of the key genera across completed validation subsets.

Genus	Completed validation subsets summarized	Random-effects pooled OR (95% CI)	I² (%)	Leave-one-out pooled OR range
Anaeroglobus	Qingdao subgroup, Guangzhou subgroup, 5-year-old cohort (partial non-independence noted)	3.335 (2.087–5.329)	0.0	3.239–3.471
Lactobacillus	Qingdao subgroup, Guangzhou subgroup, 5-year-old cohort (partial non-independence noted)	2.082 (1.310–3.308)	0.0	2.010–2.164

## Discussion

The main point of this study is not that it provides a single-genus model that can be stably applied across settings, but that it identifies a relatively consistent directional replication signal for Anaeroglobus across three saliva-based layers: the discovery stage, the primary external validation cohort, and the second independent external validation cohort (Kasimoglu et al., 2020; Li et al., 2021; Yang et al., 2023). This finding suggests that the genus has a certain probability of recurring across different pediatric cohorts, but “directional replication” is not equivalent to “model transportability” or immediate clinical usability. In the present study, the model based only on genus-level salivary 16S features still showed only moderate external AUC performance (Grier et al., 2021; Li et al., 2021; Raksakmanut et al., 2023), which is broadly consistent with the latest systematic review showing that caries risk prediction models in children commonly suffer from insufficient external validation, only moderate discrimination, and limited generalizability (Wang et al., 2025). Accordingly, the more appropriate positioning of this study is that it offers a candidate risk signal repeatedly observed across multiple cohorts, rather than claiming that a directly generalizable microbiome biomarker model has already been established. Accordingly, the current evidence supports repeated association and directional replication, not immediate clinical implementation of a transportable diagnostic model. The added approximate AUC CI improves transparency regarding discrimination but does not replace calibration or clinical-utility assessment.

From an etiologic perspective, the findings are better interpreted within a framework of caries-related ecological disequilibrium than as renewed confirmation of a single dominant cariogenic organism. Classical plaque ecology holds that caries fundamentally reflects community restructuring of the oral biofilm under the combined influence of sugar exposure, acid stress, and host microenvironment, and that the core issue is not the isolated elevation of one genus but the broader alteration of community structure, metabolic pathways, and niche competition (Marsh, 1994; Marsh, 2006; Takahashi and Nyvad, 2011). Reviews focused on ECC likewise emphasize that polymicrobial interactions and ecological functional imbalance are more informative for explaining disease onset and progression than whether one specific taxon is elevated in isolation (Simón-Soro and Mira, 2015; Hajishengallis et al., 2017). This is also in line with the cross-cohort plaque study by Khan et al., in which the more stable findings across studies were community-level differences and model-level discrimination rather than the absolute dominance of any single genus in all datasets (Khan et al., 2024). Therefore, the significance of Anaeroglobus in the present study is closer to that of a reproducible signal of cariogenic ecological shift than to independent and sufficient etiologic evidence.

The reason Anaeroglobus still merits discussion is that the signal is not devoid of biological plausibility, although this plausibility should be understood as that of an auxiliary risk marker rather than a decisive pathogen. On the one hand, prior functional-omics studies in pediatric ECC suggest that caries-associated microbiome changes are commonly accompanied by enhanced carbohydrate metabolism, acid production, and acid tolerance (Raksakmanut et al., 2023). On the other hand, plaque transcriptomic studies show that sugar utilization, disaccharide metabolism, and oxidative-stress adaptation constitute important functional backgrounds of plaque biofilms (Peterson et al., 2014). At the single-genus level, the relationship between Lactobacillus, adaptation to acidic niches, and caries progression is more clearly established (Caufield et al., 2015), whereas current evidence for Anaeroglobus more often suggests that it may influence the structure and proteomic patterns of multispecies oral biofilms (Bao et al., 2017). This implies that Anaeroglobus is more likely to be one accompanying member involved in cariogenic community restructuring, rather than an organism that can currently be defined as a core pathogen. In addition, because Anaeroglobus may be relatively low-abundance and influenced by library preparation, sequencing depth, taxonomic assignment, and CLR preprocessing, its estimated effect size may be less stable than that of well-established cariogenic taxa across different pipelines. Accordingly, the observed signal should be interpreted as part of broader dysbiosis or biofilm maturation rather than as direct evidence of an independent etiologic role. The precision check in the 36-sample cohort further reinforces that the statistically significant OR should be interpreted cautiously because the estimate remains imprecise.

The results must also be interpreted cautiously in light of both ecological niche and age-structure differences. The pediatric oral microbiome continues to evolve during growth and development, and age, dentition stage, and host behavioral exposures may all influence community composition (Gomez and Nelson, 2017; Dzidic et al., 2018). Although saliva is suitable for population screening and external validation studies, it represents a relatively integrated oral ecological signal and cannot be considered fully equivalent to the local carious lesion or plaque microenvironment (Shi et al., 2018; Hurley et al., 2019; Lee et al., 2021). Therefore, the second independent external validation cohort of 5-year-old children is better regarded as age-extrapolation support, used to test whether the signal direction can persist in a younger population, rather than being assigned exactly the same evidentiary weight as the 130-sample primary external validation cohort (Yang et al., 2023). Furthermore, the deep-caries pulp single-cell results mainly provide a background of host immune-stromal remodeling (Opasawatchai et al., 2022); their role is to indicate that host-response changes exist in the late stage of severe carious lesions, not to directly demonstrate an Anaeroglobus-specific host mechanism. This point is particularly relevant for the 36-sample 5-year-old cohort, which provides only limited statistical power and should be considered lower-level supportive evidence. Since caries-associated biofilms are localized mainly in dental plaque and lesion niches, future studies should collect paired saliva, supragingival plaque, and lesion-site samples to determine whether salivary Anaeroglobus reflects lesion-localized ecology, whole-mouth microbial spillover, or a nonspecific oral dysbiosis signal.

Overall, the main improvement of the current version over earlier drafts lies not only in the addition of a quantitative pooled analysis across completed validation subsets, but also in a clearer distinction between the “main validation results” and the “supporting evidence layer.” For Chinese core journals that value completeness, internal logic, and an interpretable chain of evidence, this structure is already able to answer two key questions reasonably well: whether a reproducible candidate signal exists, and how that signal should be interpreted. However, if the manuscript is submitted to higher-impact SCI journals, reviewers may still require unified reprocessing of raw same-ecological plaque data together with additional prospective external validation, functional validation, or multimodal integrative analyses (Peterson et al., 2014; Khan et al., 2024; Wang et al., 2025). In light of the current overall evidence from pediatric caries prediction studies (Wang et al., 2025), future work should not focus solely on continuing to search for “new single genera,” but should instead move toward building a risk-stratification framework that remains stable across ages, ecological niches, and platforms. Future validation should incorporate calibration plots, sensitivity, specificity, positive and negative predictive values, decision-curve analysis, and bootstrap or prospective validation, together with multimodal models integrating microbiome, clinical, dietary, and host-response variables.

### Limitations

First, although the present manuscript adds same-ecological plaque public-database support, plaque transcriptomic support, deep-caries pulp single-cell support, and an exploratory pooled-effect analysis, these additions do not replace unified raw-data reprocessing of plaque datasets. Individual-level original analyses such as PCoA/PERMANOVA, LEfSe, or PICRUSt2 remain to be completed. Second, the main analysis remains saliva-based. Although saliva is more practical for population studies and the twin design strengthens control of family relatedness, saliva still differs from the local lesion niche; hence same-ecological plaque evidence was added to complement this limitation. Third, the discovery and validation cohorts differed in age, dentition stage, data level, and preprocessing pipelines. The 5-year-old second external cohort included only 36 children and should therefore be interpreted as age-extrapolation support rather than as evidence equivalent to the primary external validation cohort. Fourth, the supporting evidence layer describes a general cariogenic biofilm and host-response background and cannot be used to infer a unique Anaeroglobus-specific function or host mechanism. The plaque, plaque-transcriptome, and single-cell layers were not reprocessed as unified individual-level original datasets within this manuscript and therefore remain supportive rather than primary analytical evidence. Wet-lab validation is still lacking. The 36-sample second external validation cohort remains underpowered, and its significant Anaeroglobus association should not be interpreted as equally strong evidence compared with the primary external validation cohort. In addition, the exploratory pooled-effect analysis was limited by the small number of subsets and by partial non-independence between the Qingdao and Guangzhou subsets; therefore, I²=0.0% should not be overinterpreted. Model assessment was also mainly based on AUC, and calibration and clinical utility metrics require future individual-level and prospective validation. Although an approximate AUC confidence interval was reported for the primary external model, threshold-dependent performance, calibration, and decision-curve analysis still require future individual-level validation with prespecified prediction thresholds. The back-calculated standard errors also confirmed that the 36-sample cohort was less precise than the primary validation cohort.

## Conclusions

An integrated analysis of public pediatric oral microbiome datasets showed that Anaeroglobus replicated directionally across the discovery stage, a 130-sample primary external validation cohort, and a 36-sample second independent external validation cohort, and the exploratory pooled-effect analysis descriptively suggested a pooled random-effects OR of 3.335 (95% CI: 2.087-5.329) across completed validation subsets. Lactobacillus also showed a stable pooled effect (OR = 2.082, 95% CI: 1.310-3.308), whereas the external generalizability of models based solely on salivary 16S genus-level features remained limited. The second validation cohort should be regarded as supportive age-extrapolation evidence because of its limited size. The pooled estimate should be interpreted as an exploratory synthesis of completed validation subsets rather than as definitive evidence of fully independent external reproducibility.

It should be clearly distinguished that the term “multicohort external validation” in this manuscript refers only to the discovery, primary external, and second independent external saliva validation layers. The added plaque, plaque-transcriptome, and deep-caries pulp single-cell layers belong to the supporting evidence layer and serve only to indicate that the observed signal is more plausibly situated within a broader background of plaque community restructuring, enhanced carbohydrate metabolism and oxidative-stress adaptation, and host immune-stromal remodeling. Accordingly, Anaeroglobus is better interpreted as a candidate risk-related auxiliary signal warranting further validation, rather than as an immediately translatable single-genus diagnostic tool.

## Data Availability

The original contributions presented in the study are included in the article/supplementary material. Further inquiries can be directed to the corresponding authors.
